# Response of Mungbean (cvs. Celera II-AU and Jade-AU) and Blackgram (cv. Onyx-AU) to Transient Waterlogging

**DOI:** 10.3389/fpls.2021.709102

**Published:** 2021-08-19

**Authors:** Khin Lay Kyu, Al Imran Malik, Timothy David Colmer, Kadambot H. M. Siddique, William Erskine

**Affiliations:** ^1^Centre for Plant Genetics and Breeding (PGB), University of Western Australia (UWA) School of Agriculture and Environment, Perth, WA, Australia; ^2^UWA Institute of Agriculture, The University of Western Australia, Perth, WA, Australia

**Keywords:** mungbean, blackgram, waterlogging, adventitious roots, germination

## Abstract

Mungbean [*Vigna radiata* (L.) Wilczek] and blackgram [*Vigna mungo* (L.) Hepper] are important crops for smallholder farmers in tropical and subtropical regions. Production of both crops is affected by unexpected and increasingly frequent extreme precipitation events, which result in transient soil waterlogging. This study aimed to compare the waterlogging tolerance of mungbean and blackgram genotypes under the varying duration of waterlogging stress at germination and seedling stages. We evaluated the responses to different durations of transient waterlogging in a sandy clay loam under temperature-controlled glasshouse conditions. Waterlogging durations were 0, 1, 2, 3, 4, 5, 6, 7, and 8 days during germination and 0, 2, 4, 8, and 16 days during the seedling stage. We used two mungbean genotypes (green testa), Celera II-AU (small-seeded), and Jade-AU (large-seeded), contrasting in seed size and hypocotyl pigmentation, and a blackgram genotype (black testa), Onyx-AU. Waterlogging reduced soil redox potential, delayed or even prevented germination, decreased seedling establishment, and affected shoot and root development. In the seedlings waterlogged (WL) at 15 days after sowing (DAS), adventitious root formation and crown nodulation varied between the genotypes, and 16 days of waterlogging substantially reduced growth but did not result in plant death. Plants in soil with waterlogging for 8–16 days followed by drainage and sampling at 39 DAS had reduced shoot and root dry mass by 60–65% in mungbean and 40% in blackgram compared with continuously drained controls, due at least in part to fewer lateral roots. Soil plant analysis development (SPAD) chlorophyll content was also reduced. Onyx-AU, a blackgram genotype, was more tolerant to transient waterlogging than Jade-AU and Celera II-AU in both growth stages. Of the two mungbean genotypes, Celera II-AU had a greater seedling establishment than Jade-AU post waterlogging imposed at sowing. In contrast, Jade-AU had more plant biomass and greater recovery growth than Celera II-AU after waterlogging and recovery during the seedling stage. Both species were delayed in emergence in response to the shorter periods of transient waterlogging at germination, and with the longer waterlogging germination and emergence failed, whereas at the seedling stage both showed adaptation by the formation of adventitious roots.

## Introduction

Mungbean [*Vigna radiata* (L.) Wilczek] and blackgram [*Vigna mungo* (L.) Hepper] are short-season (sub)tropical grain legumes and are important due to their valuable seed nutritional composition for the human diet and income for growers (Somta and Srinives, 2007). The crops are mainly grown in Asia with some cultivated in Africa and Oceania. Globally, mungbean covers more than 7.3 million ha with an annual global production of 5.3 million tons. India and Myanmar each produce about 30% of global output (Nair and Schreinemachers, 2020). Global blackgram production reached 3.2 million tons in 2018, with India producing 1.9 million tons on 3.5 million ha and Myanmar generating 1.24 million tons on 9.78 million ha (Soe et al., 2020). Both crops are predominantly grown in the tropics in rainfed farming systems (Lawn and Ahn, 1985), where yield variation is high due to biotic and abiotic stresses.

Abiotic stresses are a major environmental problem in agricultural crop production (Lesk et al., 2016). Soil waterlogging is abiotic stress that can affect crop growth and development. This adversely affects crop production and the profits of farmers. In waterlogged (WL) soil, oxygen deprivation is the major impediment to root growth and functioning. Oxygen is consumed by the respiration of plant roots and soil microorganisms so the soil becomes anoxic within a few hours to days (Ponnamperuma, 1984; Setter and Waters, 2003). Oxygen deficiency in WL soil can adversely affect nutrient uptake and translocation by roots; as an example, Malik et al. (2002) demonstrated in wheat that shoot N status was reduced during the first 7 days of waterlogging that eventually affected growth. Rasaei et al. (2012) reported that soil N content decreases through rapid volatilization and denitrification processes. Furthermore, prolonged waterlogging can lead to the accumulation of some ions (i.e., Mn^2+^, Fe^2+^) to potentially toxic levels (McKee and McKevlin, 1993). In these conditions lacking oxygen, oxidative phosphorylation ceases, yielding low ATP from sugar catabolism and hindering the metabolic functions required for seed germination and then for seedlings for root growth and nutrient acquisition by roots (Yamauchi et al., 2018).

Mungbean and blackgram are both considered highly sensitive to soil waterlogging, mainly during the early stages of growth (Bansal et al., 2019; Douglas et al., 2020). Crops can often be exposed to transient waterlogging during their growth cycle due to extreme weather events (i.e., intense storms bringing rain) and poor soil drainage. Fernandez and Shanmugasundaram (1988) reported that mungbean yields severely declined with annual rainfall >1,000 mm. Flooding restricts the aeration around mungbean roots reducing nodule activity and N fixation (Singh and Singh, 2011). In addition, the “weakened” plants can be further infected by fungal diseases and suffer from insect pests (Tickoo et al., 2006).

Mungbean and blackgram are widely grown in both upland and lowland ecosystems in Asia. In upland ecosystems of Southeast and South Asia, mungbean is grown as an intercrop with other legumes such as pigeonpea (*Cajanus cajan* L.), oilseeds [sesame (*Sesamum indicum* L.), and groundnut (*Arachis hypogaea* L.)], or cereal crops [Sorghum (*Sorghum bicolor* L.), and maize (*Zea mays* L.)] during pre-monsoon and monsoon seasons (Islam et al., 1993; Herridge et al., 2019) and blackgram is sown as a sole crop during the post-monsoon period. In lowland ecosystems, both legumes are widely grown as relay crops by broadcasting onto the standing rice crop 7–10 days before harvest or dibbling manually after harvest (Gupta et al., 2016). Excess soil moisture immediately before or after rice harvest exposes the seeds of the succeeding crop to waterlogging stress, resulting in reduced germination and/or poor crop establishment, as documented for field and glasshouse experiments for different legumes (Zaman et al., 2018). This type of waterlogging stress has been observed in some grain legumes grown as relay crops after rice and studied for their waterlogging tolerance—for instance, pea (*Pisum sativum* L.), lentil (*Lens culinaris* L.), grass pea (*Lathyrus sativus* L.), and soybean (*Glycine max* L.)—in countries in South, Southeast, and East Asia, including Bangladesh, India, Nepal, Pakistan, and Japan (Samad et al., 2001; Araki, 2006; Malik et al., 2015; Zaman et al., 2018).

To overcome crop yield limitations due to waterlogging stress, it is essential to understand the tolerance mechanism of crops to the stress and develop climate-resilient varieties. Plants adapted to complete submergence which occurs from deep floods but also can occur for seeds and seedlings in shallow water and WL soils, have two syndromes (groups of mechanisms) for coping with waterlogging, namely, quiescence and escape (Bailey-Serres and Voesenek, 2008; Colmer and Voesenek, 2009). Recently, both syndromes have been observed in pea genotypes at germination: quiescence was characterized as no germination during several days of waterlogging and following soil drainage germination occurred and seedlings emerged, whereas escape was germination and seedling emergence during waterlogging (Zaman et al., 2018). Furthermore, testa integrity of seed under waterlogging is a key tolerance trait for germination (Zaman et al., 2019) because seed testa serves as a shield for the embryo against adverse environments (Debeaujon et al., 2000). Germinating seeds can tolerate anoxia (absence of oxygen) after imbibition but before the rupture of the testa (Leblová et al., 1969). Rupturing seed testa due to waterlogging leads to deterioration of membranes and leakage of cellular contents, failing germination, and/or seed death (Johnson et al., 1989; Zaman et al., 2019). Information on the physiological responses of mungbean to soil waterlogging is scarce, and the types of waterlogging tolerance mechanisms exhibited by mungbean and blackgram remain unknown, especially in early growth (Douglas et al., 2020). Therefore, this study focused on the waterlogging tolerance of mungbean and blackgram genotypes under the varying duration of waterlogging stress at two critical growth stages, such as germination and seedling stages.

## Materials and Methods

### Plant Materials

Two mungbean genotypes, Celera II-AU and Jade-AU, with contrasting seed size and hypocotyl pigmentation, and blackgram genotype, Onyx-AU, were used in this study. A single blackgram genotype was used as a benchmark for the two genotypes of mungbean, which is of greater economic importance. The genotypes were obtained from the Department of Agriculture and Fisheries (DAF), Queensland (Table 1).

**Table 1:** Hypocotyl pigmentation and testa color, 100 seed weight, and days to flowering and maturity in two mungbean genotypes (Celera II-AU and Jade-AU) and a blackgram genotype (Onyx-AU).

**Cultivar**	**Species**	**Hypocotyl pigmentation**	**Seed testa color**	**100 seed weight (g)**	**Days to**	
					**Flower**	**Maturity**
Celera II-AU	*V. radiata*	Purple	Shiny green	4.2	42	77
Jade-AU	*V. radiata*	Green	Shiny green	8.1	44	85
Onyx-AU	*V. mungo*	Purple	Black	6.6	45	>80

*Source: Australian Mungbean Association (AMA) (2020)*.

### Experimental Conditions

The experiments were conducted in a temperature-controlled glasshouse at the University of Western Australia (UWA), Crawley, Western Australia (31°59′ S, 115°49′ E) from 15 May to 7 June 2018 (germination stage) and from 10 October to 19 November 2018 (seedling stage). The temperature inside the glasshouse ranged from 21 ± 4 (night) to 32 ± 3°C (day) in both growth stages. The day length was ~10 h 20 min with maximum PAR of 1,000–1,098 μmol m^−2^ s^−1^ in May and 12–14 h day length in October–November with PAR of 1,400–1,630 μmol m^−2^ s^−1^. The growing media comprised red-brown sandy clay loam (Calcic Haploxeralf), which had been used for waterlogging studies in pea (Zaman et al., 2018) and grass pea (Wiraguna et al., 2020). The soil was collected from Mukinbudin (30°78′ S, 118°31′ E), Western Australia (Kotula et al., 2015), with soil pH (CaCl_2_) of 7.8, electrical conductivity (EC) 0.64 dS m^−1^, and 1:5 w/v soil/water organic carbon content of 0.26%. The soil was dried for 5 days at 65°C and sieved to 2 mm diameter. The water content (w/w) at field capacity (i.e., pot capacity when fully drained) was 18%.

### Experiment A: Waterlogging at Germination Stage

The experimental design was a split-plot with three replications. Waterlogging was the main factor and genotypes were subfactors. The genotypes (Table 1) were exposed to nine durations of waterlogging (0, 1, 2, 3, 4, 5, 6, 7, or 8 days). At the end of the waterlogging period, WL pots were drained and seedling emergence was recorded. The experiment ended 15 days after sowing (DAS).

Seeds were surface sterilized with 1% commercial bleach (active ingredients NaOCl 40 mg L^−1^) for 1 min and rinsed with deionized (DI) water four times. To control seed-borne and seedling root pathogens, P-Pickel T liquid fungicide [Thiram (360 g L ^−1^) + Thiabendazole (200 g L^−1^)] was applied 300 ml 100 kg^−1^ seed. Twenty seeds of each genotype in each of 48 pots were sown either in drained (control) or WL pots at 10 mm depth. The experimental pots were 0.8 L (90 mm × 90 mm × 180 mm) with drainage holes (~10 mm) at the bottom. The drainage holes were covered with filter paper to avoid soil loss, and the pots were filled with 100 g of gravel followed by 1.0 kg of soil. After potting up, all the pots were placed in 60 L plastic tanks. Then the pots were kept at 80% field capacity for 2 days before sowing and layout according to the design. Twelve platinum electrodes: six each for drained and for WL pots were placed at 100 mm depth to measure soil redox potential.

Waterlogging treatment was imposed immediately after sowing by adding DI water to the 60 L tanks as described by Zaman et al. (2018). The water table was maintained at the soil surface for the duration of the waterlogging treatment by adding DI water to the tanks. Drained control pots were kept at 80% field capacity by adding DI water directly to the pots as required throughout the experimental period. At the end of each waterlogging treatment, pots were relocated to free-draining plastic tanks to record emergence and seedling growth during the recovery. The 80% field capacity was maintained in the recovery pots by adding additional DI water as required. After 15 days of recovery, the germinated seedlings were gently washed from the soil with tap water for measurements.

### Measurements for Experiment A

Soil redox potential was measured daily with platinum electrodes (Pt) and an Ag–AgCl reference electrode using a handheld Digital Multimeter (Fluke 114, Everett, Washington, USA). Redox measurements were corrected according to the method developed by Patrick et al. (1996). Seeds with an epicotyl >5 mm were recorded as germinated (i.e., emergence). At harvest, the percentage of the seedling establishment was recorded based on the number of fully grown seedlings.

### Experiment B: Waterlogging at Seedling Stage

The experiment had a split-plot design with four replications. Duration of waterlogging [0 (WL0), 2 (WL2), 4 (WL4), 8 (WL8), and 16 (WL16) days] was the main factor, and genotype (Table 1) was the subfactor. After waterlogging, the WL pots were drained to observe plant growth during the recovery. The duration of the recovery period differed for each waterlogging treatment (WL2: 22 days, WL4: 20 days, WL8: 16 days, and WL16: 8 days), with the experiment terminated 39 DAS. Six Pt electrodes were placed at 100 mm depth in each WL and drained soil to measure soil redox potential.

Seeds were surface sterilized as described for Experiment A and then inoculated with Group I Rhizobium strain CB 1015 (New Edge Microbial, New South Wales, Australia). Six seeds per pot [free draining 4 L plastic pots (145 mm × 145 mm × 220 mm) with drainage holes (15 mm)] were sown at 30 mm depth in 76 pots for each genotype. Four days after emergence, plants were thinned to two seedlings per pot with similar vigor. The drainage holes inside the pots were covered with filter paper, before filling with 500 g of gravel followed by 4 kg of sieved dry soil. The pots were placed in 60 L (310 mm × 620 mm × 455 mm) plastic tanks (eight pots per tank) and watered as necessary to keep at 80% field capacity. Each pot was an experimental unit and received 40 mg kg^−1^ of dihydrogen ammonium phosphate [(NH_4_) (H_2_PO_4_)]; this level was based on a soil analysis.

Waterlogging treatments were imposed 15 DAS, which was after the first trifoliate leaf had fully opened. For each genotype, 56 pots were WL to the soil surface, with 20 kept as drained controls watered daily to 80% of field capacity. The harvesting methodology was similar to that used by Malik et al. (2002). Considering the start of the treatment as Day 0 (H1), four pots from each genotype were harvested as initial harvest, with sequential harvesting on Days 2, 4, 8, 16, and 24 (Table 2). On Day 2 (H2), four control pots and four WL pots from each genotype were harvested; meanwhile, 16 WL pots were drained for harvest on Days 4, 8, 16, and 24. Similarly, four control pots and four WL pots were harvested on Day 4 (H3), with 12 pots drained for harvest on Days 8, 16, and 24. On Day 8 (H4), four from control pots and four WL pots were harvested, with eight pots drained for harvest on Days 16 and 24. On Day 16 (H5), four control pots and four WL pots were harvested, with four pots drained for harvest on Day 24 (H6). This sequential harvest increased the number of treatments at each successive harvest, resulting in six experimental treatments at the final harvest on Day 24.

**Table 2:** Harvesting schedule for Experiment B: Initial harvest occurred on the day waterlogging was imposed at 15 days after sowing (DAS).

**Treatment**	**Harvest**	**DAS**
WL0	H1 (Day 0)	15
WL0, WL2	H2 (Day 2)	17
WL0, WL2, WL4	H3 (Day 4)	19
WL0, WL2, WL4, WL8	H4 (Day 8)	23
WL0, WL2, WL4, WL8, WL16	H5 (Day 16)	31
WL0, WL2, WL4, WL8, WL16	H6 (Day 24)	39

*Waterlogging lasted for a maximum of 16 days. Sequential harvests occurred for each waterlogging duration treatment with successive recovery compared with the drained controls*.

### Measurements for Experiment B

Soil redox potential was measured daily in the WL and drained pots as described for Experiment A. The number of large and small, fully opened trifoliate leaves of one plant per pot was recorded the day before harvest. At harvest, parameters were recorded on two plants per pot. The data for each pot (2 plants in each pot) were pooled and the mean was used as one replicate.

Chlorophyll content was measured using a handheld Minolta SPAD 502 (Konica-Minolta, Japan) on the first trifoliate leaf of each plant on the day of harvest. Twelve independent measurements per genotype were done for each treatment. At harvest, plant height was measured from the collar (point on the stem where roots start to grow) to the leaf base of the youngest fully expanded leaf of the plant. After gently washing the soil from the roots, the maximum taproot length was measured. The number of emerged adventitious roots longer than 5 mm were recorded, and their length recorded. Total leaf area per plant was recorded for each plant using a leaf area meter (LI 3000C, Lincoln NE, USA). Nodulation was scored by counting the nodules on the main taproot and lateral roots and using a 0–8 scoring scale according to Yates et al. (2016), where 0 = no nodules, 0.5 = white ineffective nodules, 1 = rare effective, 2 = scarce, 3 = moderate, 4 = adequate, 5 = ample, 6 = abundant, 7 = very abundant, and 8 = extremely abundant. Finally, the plants were divided into shoots and roots and dried in paper bags in a 60°C oven for 3 days to record shoot and root dry weights.

### Statistical Analysis

ANOVA was performed for each waterlogging treatment and compared with its controls using GenStat 19th edition (VSN International, UK). The effect of waterlogging was based on the significance level of main and interaction effects. For all analyses, the means were separated based on their significance levels at 0.05 probability using the Tukey test (Tukey, 1949). The estimated percent SPAD chlorophyll content was based on the untreated mean. The relative growth rate (RGR) of shoots and roots was calculated for each waterlogging duration and their successive recovery period according to Hunt (1990).

## Results

### Waterlogging Tolerance at Germination Stage

The redox potential in WL pots decreased gradually from 428 ± 18 mV to 236 ± 38 mV, stabilizing after 5 days of waterlogging (Supplementary Figure 1). The drained control pots remained at 428 ± 18 mV throughout the experimental period.

**Figure 1:**
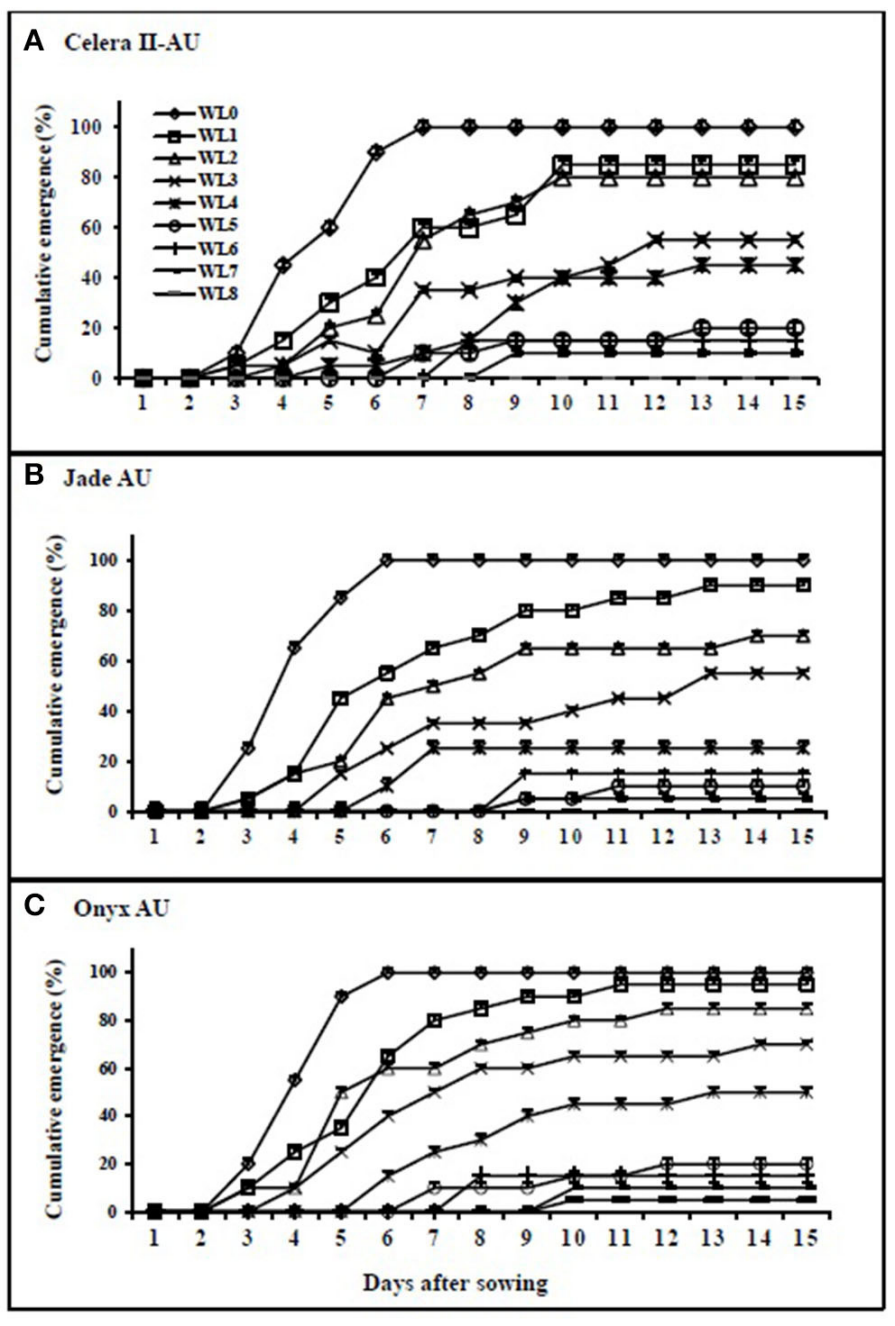
Percentage of cumulative emergence in mungbean **(A)** Celera II-AU and **(B)** Jade-AU and blackgram **(C)** Onyx-AU under different durations of waterlogging and subsequent recovery. WL0, drained control; WL1, waterlogging for 1 day and recovery for 14 days; WL2, waterlogging for 2 days and recovery for 13 days; WL3, waterlogging for 3 days and recovery for 12 days; WL4, waterlogging for 4 days and recovery for 11 days; WL5, waterlogging for 5 days and recovery for 10 days; WL6, waterlogging for 6 days and recovery for 9 days; WL7, waterlogging for 7 days and recovery for 8 days; WL8, waterlogged (WL) 8 days and recovery for 7 days. Symbols are means ± SE of three replicates.

Waterlogging reduced seedling emergence in all genotypes, relative to the drained control (Figure 1). The WL seedlings started to emerge 2 days after removing the stress, with full emergence within 10 days after removal of the stress on average. Emergence in the drained control pots started three DAS, with full emergence completed by seven DAS in all genotypes. Seedling emergence was significantly (*P* < 0.001) reduced by the waterlogging duration.

**Figure 2:**
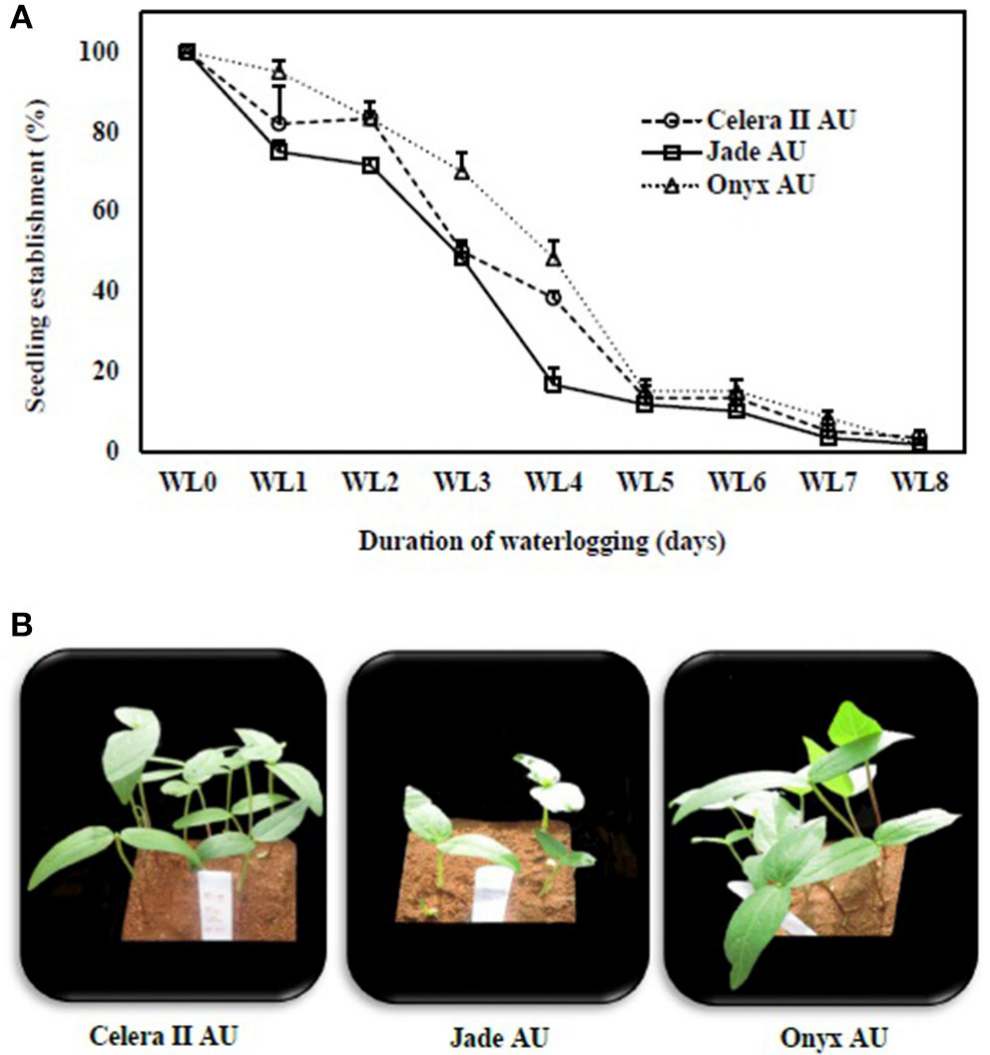
Seedling establishment (%) of **(A)** mungbean (°) Celera II-AU, (□) Jade-AU and blackgram (Δ) Onyx-AU after different durations of waterlogging (WL0, drained control; WL1, waterlogging for 1 day; WL2, waterlogging for 2 days; WL3, waterlogging for 3 days; WL4, waterlogging for 4 days; WL5, waterlogging for 5 days; WL6, waterlogging for 6 days; WL7, waterlogging for 7 days; WL8, waterlogging for 8 days) and subsequent recovery days. Symbols are the mean ± SE of three replicates. **(B)** Photographs of fully emerged seedlings of the same genotypes after 4 days of waterlogging and subsequent 8 days of recovery.

With the 4 days of WL treatment, the largest genotypic differences were observed for the number of seedlings, with Onyx-AU at 50% followed by Celera II-AU at 45% and Jade-AU at 25% (Figure 2A). Some WL seeds failed to emerge; for example, only 5% of Onyx-AU seedlings had emerged in the 8 days of waterlogging treatment by the end of the experiment, with none for the other two genotypes (Figure 2B).

### Waterlogging Tolerance at Seedling Stage

#### Soil Redox Potential

**Figure 3:**
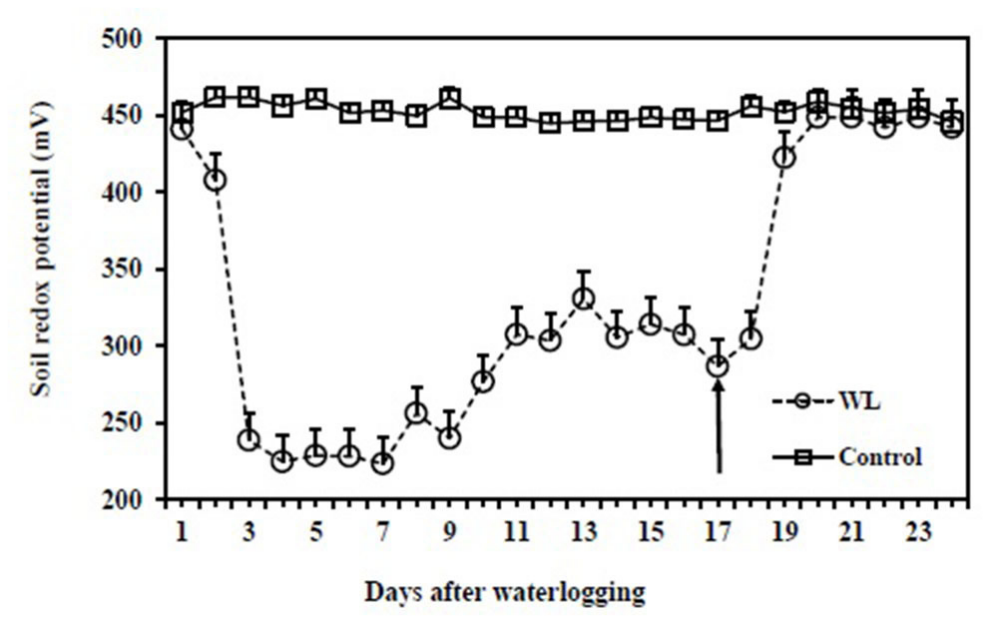
Comparison of the soil redox potential of drained control and continuous waterlogging for 16 days followed by recovery upon drainage. The arrow represents the first day of recovery after the release of the waterlogging treatment. The vertical bars represent the means ± SE.

At the start of the waterlogging treatment, soil redox potential in the drained control pots was 452 ± 9 mV where it remained throughout the experiment (Figure 3). During waterlogging, soil redox potential rapidly decreased to 239 ± 14 mV on Day 2, plateauing at 225 ± 13 mV on Day 3, where it remained until Day 7, before increasing gradually to 330 ± 25 mV by Day 13. This increase in redox potential coincided with an increasing number of adventitious roots (as shown below). Once the treatment was completed on Day 16, the WL pots were drained to allow the plants to recover. At recovery, the soil redox potential returned to the control value (459 ± 5 mV) within 4 days.

### Shoot Growth

Soil waterlogging strongly adversely affected seedling growth. The ANOVA showed that the duration of waterlogging had a higher mean square than genotype and the interaction for all parameters except nodulation score and maximum taproot depth (Table 3). The effects of waterlogging duration, genotype, and their interaction were significant for all characters.

**Table 3:** Degrees of freedom (df), *F*-values, and probabilities of two-way ANOVA at the seedling stage.

**Character**	**Source of variation**	**Treat**	**Genotype**	**G × T**
		**(T)**	**(G)**	
	df	19	2	38
SPAD chlorophyll content	*F*-value	79.1	55.36	2.02
	Probability	<0.001	<0.001	0.002
Leaf area per plant	*F*-value	75.16	37.48	4.77
	Probability	<0.001	0.004	<0.001
No. trifoliate leaves	*F*-value	106.04	29.63	3.87
	Probability	<0.001	<0.001	<0.001
Plant height (cm)	*F*-value	65.50	23.45	3.36
	Probability	<0.001	<0.001	<0.001
Shoot dry weight (g)	*F*-value	119.3	51.9	7.05
	Probability	<0.001	<0.001	<0.001
Root dry weight (g plant^−1^)	*F*-value	80.16	47.51	4.32
	Probability	<0.001	<0.001	<0.001
Total dry weight (g plant^−1^)	*F*-value	128.82	51.72	7.50
	Probability	<0.001	<0.001	<0.001
Nodulation score	*F*-value	7.42	20.21	2.3
	Probability	<0.001	<0.001	<0.001
Taproot depth (cm)	*F*-value	5.08	17.79	2.54
	Probability	<0.001	<0.001	<0.001
No. adventitious roots	*F*-value	104.7	13.4	1.95
	Probability	<0.001	<0.001	<0.001

**Figure 4:**
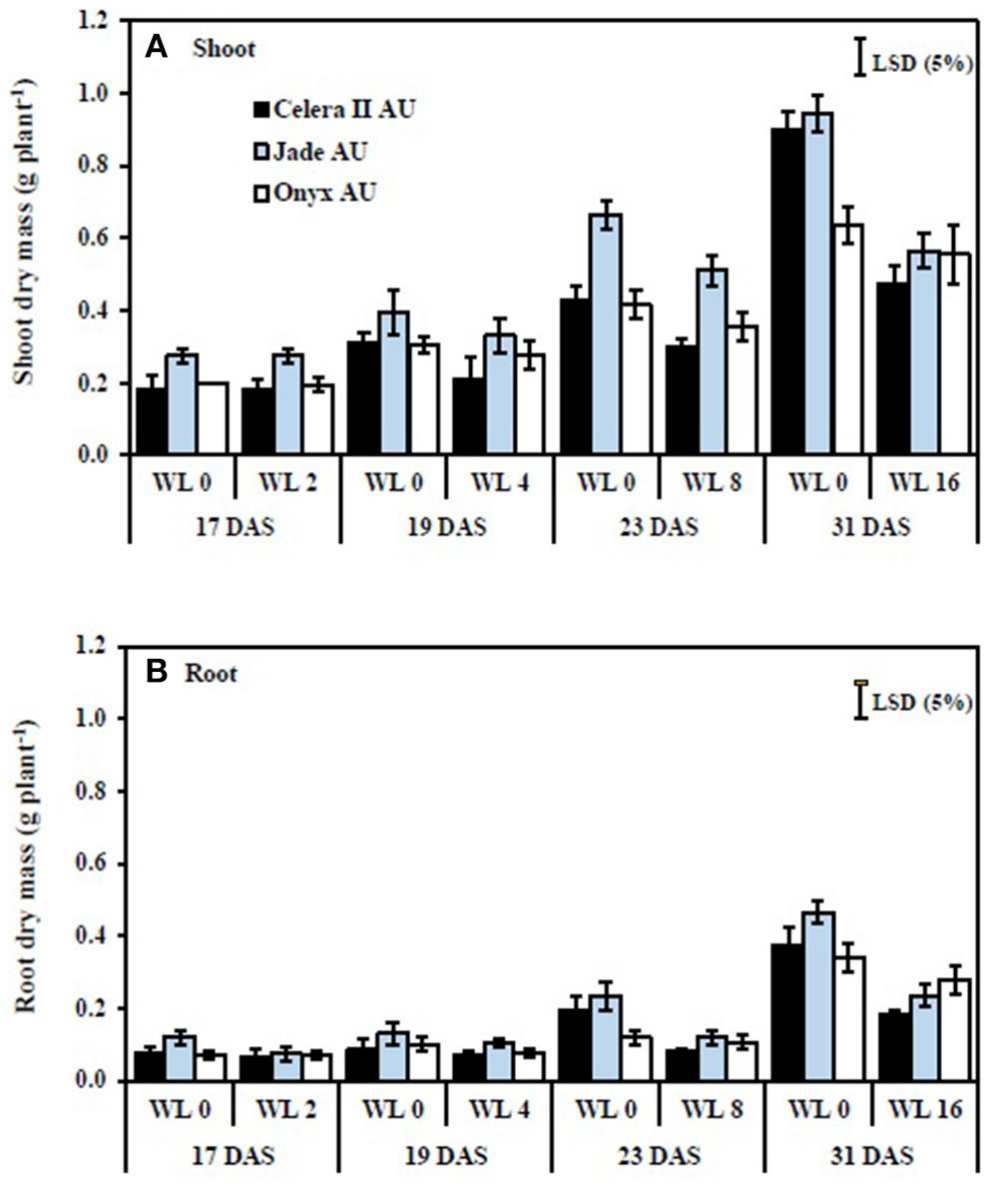
The effect of different waterlogging durations on the shoot **(A)** and root **(B)** dry mass of three genotypes grown in soil: WL0, drained control; WL2, WL for 2 days, harvested at 17 days after sowing (DAS); WL4, WL for 4 days, harvested at 19 DAS; WL8, WL for 8 days, harvested at 23 DAS; WL16, WL for 16 days, harvested at 31 DAS. Treatments were imposed at 15 DAS. Bars are means (*n* = 4) ± SE and least significant differences (LSD) at *P* = 0.05 for genotype.

**Figure 5:**
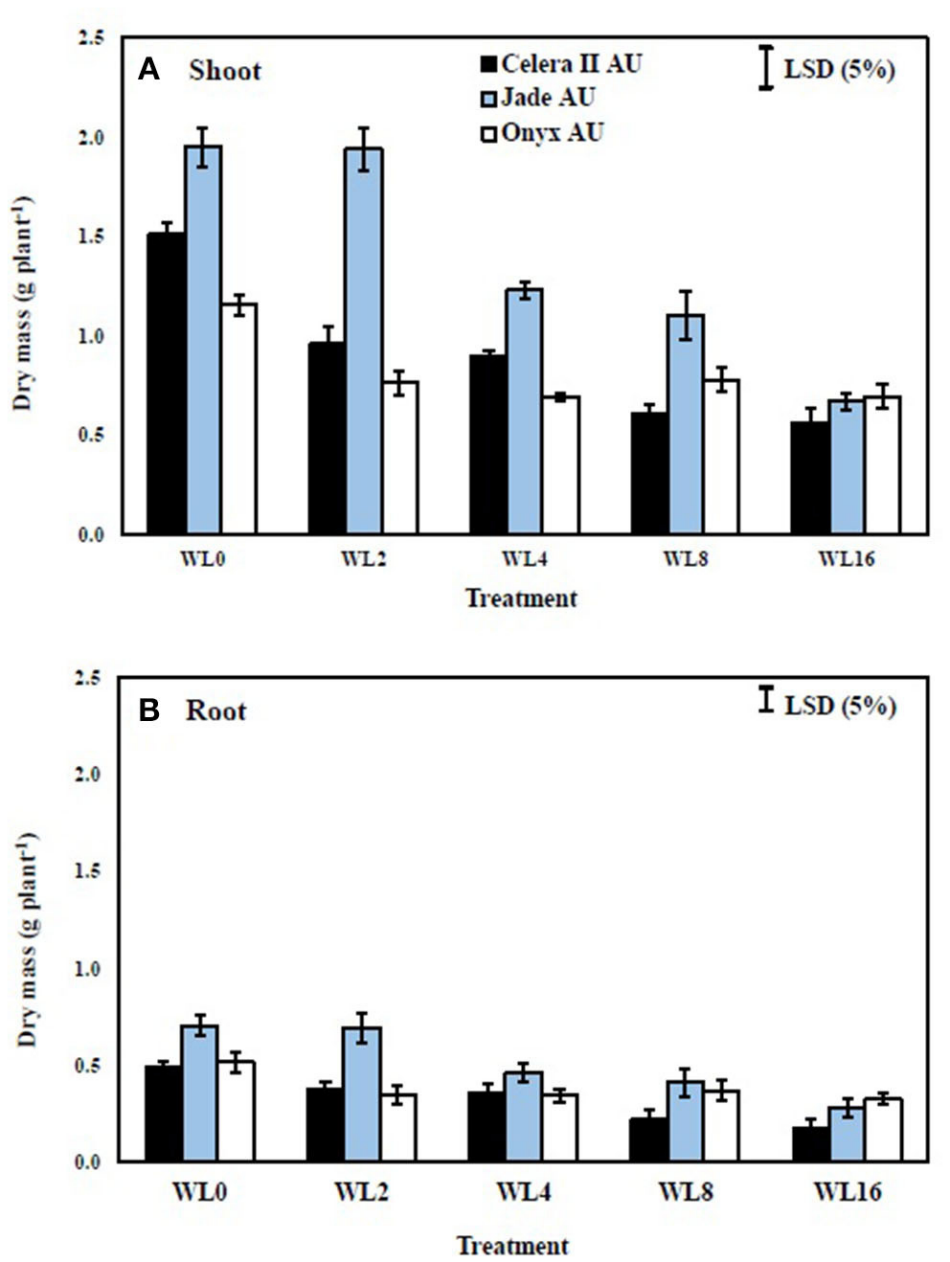
The effect of different waterlogging durations on the shoot **(A)** and root **(B)** dry mass of three genotypes after different durations of recovery at 39 DAS: WL2, waterlogging for 2 days, recovery for 22 d; WL4, waterlogging for 4 days, recovery for 20 days; WL8, waterlogging for 8 days, recovery for 16 days; WL16, waterlogging WL 16 days, recovery for 8 days. Bars are mean (*n* = 4) ± SE and LSD at *P* = 0.05 for genotype.

In the drained control soil (39 DAS), the large-seeded mungbean Jade-AU had 22% more shoot dry mass than the small-seeded mungbean Celera II-AU and 40% more than blackgram Onyx-AU. During waterlogging, shoot growth reductions were similar in both species, with maximum reductions of 47% in Celera II-AU, 40% in Jade-AU, and 41% in Onyx-AU, relative to the drained controls after 16 days of waterlogging (Figure 4A). Shoot growth declined more during recovery than during waterlogging (Figure 5A). At the end of the recovery period (39 DAS), the drained controls had 1–3 times more shoot dry mass than the WL plants. Nevertheless, waterlogging for 2 days did not affect the plant growth of Jade-AU (Figures 5A,B). Among genotypes, the growth of Celera II-AU declined the most for every waterlogging treatment (Figure 5A) at the final harvest (39 DAS). In contrast, Onyx-AU had the potential to recover its growth from the damage of waterlogging stress during the subsequent period of drainage.

The effects of waterlogging on other growth-related traits, such as plant height and the number of trifoliate leaves, are shown in Tables 4, 5. Waterlogging did not significantly reduce plant height in the earlier days of treatment, and 16 days of waterlogging (WL16) treatment produced shorter (by 19–20%) plants than those in the drained control.

**Table 4:** Plant height, number of trifoliate leaves, taproot depth, and nodulation score after different waterlogging durations.

**Waterlogging treatment**	**Plant height (cm)**		**No. of trifoliate**		**Max. depth of**		**Nodulation score**	
			**leaves**		**tap root (cm)**			
	**WL0**	**WL**	**WL0**	**WL**	**WL0**	**WL**	**WL0**	**WL**
**WL2 (17 DAS)**								
Celera II-AU	15 ± 0.5	15 ± 0.3	1 ± 0.2	1 ± 0.1	28 ± 0.3	27 ± 1.2	2 ± 0.2	1 ± 0.2
Jade-AU	15 ± 0.2	14 ± 0.2	1 ± 0.2	1 ± 0.2	32 ± 0.4	31 ± 0.7	4 ± 0.2	4 ± 0.1
Onyx-AU	12 ± 0.2	12 ± 0.2	1 ± 0.1	1 ± 0.1	31 ± 0.3	31 ± 1.0	4 ± 0.2	4 ± 0.2
**WL4 (19 DAS)**								
Celera II-AU	18 ± 0.2	17 ± 0.8	2 ± 0.2	2 ± 0.1	32 ± 1.8	32 ± 1.8	4 ± 0.3	3 ± 0.5
Jade-AU	15 ± 0.5	14 ± 0.2	2 ± 0.1	1 ± 0.2	35 ± 1.9	32 ± 0.9	5 ± 0.6	4 ± 0.5
Onyx-AU	15 ± 0.2	15 ± 0.3	2 ± 0.1	1 ± 0.2	35 ± 1.8	32 ± 0.8	6 ± 0.4	4 ± 0.2
**WL8 (23 DAS)**								
Celera II-AU	20 ± 0.6	18 ± 0.6	2 ± 0.1	2 ± 0.2	33 ± 0.2	32 ± 0.9	4 ± 0.5	3 ± 0.2
Jade-AU	17 ± 0.3	17 ± 0.5	2 ± 0.2	2 ± 0.1	37 ± 0.2	32 ± 1.7	6 ± 0.4	4 ± 0.4
Onyx-AU	18 ± 0.2	16 ± 0.1	2 ± 0.2	2 ± 0.1	28 ± 1.6	28 ± 1.1	6 ± 0.2	5 ± 0.2
**WL16 (31 DAS)**								
Celera II-AU	23 ± 0.9	20 ± 0.8	4 ± 0.1	3 ± 0.2	36 ± 0.2	28 ± 0.2	6 ± 0.4	4 ± 0.3
Jade-AU	21 ± 1.1	18 ± 0.5	4 ± 0.2	3 ± 0.1	37 ± 0.2	36 ± 0.9	6 ± 0.3	4 ± 0.4
Onyx-AU	20 ± 0.5	17 ± 0.3	4 ± 0.2	3 ± 0.1	37 ± 0.7	30 ± 0.6	6 ± 0.3	5 ± 0.4
LSD (5%) G		0.7		0.1		1.4		0.5
LSD (5%) T		1.2		0.1		3.0		0.7
LSD (5%) G × T		2.1		0.2		4.3		1.3

*W0, drained control; WL, waterlogging; WL2, waterlogging for 2 days; WL4, waterlogging for 4 days; WL8, waterlogging for 8 days; WL16, waterlogging for 16 days. Data show mean (± SE) of four replicate pots and LSD at P = 0.05 for the genotype (G), treatment (T), and genotype × treatment interaction (G × T). On Day 0, two plants were harvested in each replicate. Nodulation was scored by counting the nodules on the main taproot and lateral roots and using a 0–8 scoring scale according to Yates et al. (2016), where 0 = no nodules, 0.5 = white ineffective nodules, 1 = rare effective, 2 = scarce, 3 = moderate, 4 = adequate, 5 = ample, 6 = abundant, 7 = very abundant, and 8 = extremely abundant*.

**Table 5:** Plant height, number of trifoliate leaves, maximum depth taproot depth, and nodulation score at the end of the recovery, as affected by the duration of transient waterlogging.

**Waterlogging**	**Plant height**	**No. of trifoliate**	**Max. depth of**	**Nodulation**
**treatment**		**leaves**	**tap root (cm)**	**score**
**Celera II-AU**				
W0	25 ± 0.9	4 ± 0.2	39 ± 0.4	6 ± 0.6
WL2	22 ± 0.6	4 ± 0.1	33 ± 0.5	5 ± 0.2
WL4	23 ± 0.5	3 ± 0.1	33 ± 1.3	5 ± 0.3
WL8	21 ± 0.4	3 ± 0.1	31 ± 1.6	4 ± 0.3
WL16	21 ± 0.6	3 ± 0.2	28 ± 1.3	4 ± 0.6
**Jade-AU**				
W0	29 ± 1.1	4 ± 0.1	38 ± 2.5	7 ± 0.2
WL2	27 ± 0.2	3 ± 0.2	33 ± 1.8	5 ± 0.3
WL4	24 ± 0.9	3 ± 0.2	30 ± 1.6	5 ± 0.4
WL8	23 ± 1.5	3 ± 0.2	33 ± 0.8	4 ± 0.2
WL16	21 ± 0.1	3 ± 0.2	36 ± 1.6	5 ± 0.2
**Onyx-AU**				
W0	25 ± 0.5	6 ± 0.2	38 ± 2.1	7 ± 0.2
WL2	20 ± 1.1	5 ± 0.2	33 ± 2.2	4 ± 0.2
WL4	20 ± 0.5	5 ± 0.2	30 ± 0.4	5 ± 0.2
WL8	23 ± 0.3	5 ± 0.2	32 ± 1.3	5 ± 0.5
WL16	23 ± 0.5	4 ± 0.2	37 ± 0.4	4 ± 0.2
LSD (5%) G	0.51	0.12	14.62	0.3
LSD (5%) T	1.32	0.31	6.98	0.8
LSD (5%) G × T	2.30	0.54	2.09	1.3

*WL0, drained control; WL2, waterlogging for 2 days, recovery for 22 days; WL4, waterlogging for 4 days, recovery for 20 days; WL8, waterlogging for 8 days, recovery for 16 days; WL16, waterlogging for 16 days, recovery for 8 days. Data show mean (± SE) of four replicate pots and LSD at P = 0.05 for the genotype (G), treatment (T), and genotype × treatment interaction (G × T). On Day 0, two plants were harvested in each replicate*.

Waterlogging also affected the total leaf area. The two treatments (WL8 and WL16) reduced total leaf area in Celera II-AU, Jade-AU, and Onyx-AU by 40, 22, and 21%, respectively, relative to the controls. Despite Celera II-AU producing new leaves during waterlogging, it had a smaller total leaf area than the other genotypes, signifying its sensitivity to waterlogging (Supplementary Figure 2A). During the recovery, leaf area expansion of Celera II-AU ceased but slightly increased in Jade-AU and Onyx-AU (Supplementary Figure 2B).

### Root Growth

The taproot and lateral roots in the drained soil reached the base of pots at the start of waterlogging treatment [i.e., initial harvest (H1)]. Waterlogging reduced root system size in both species, more so with longer durations of waterlogging. Furthermore, waterlogging duration had a highly significant (*P* < 0.001) effect on root dry mass for all three genotypes (Table 3). At the end of the waterlogging period, the WL8 treatment had reduced root dry matter in Celera II-AU by 26% and Jade-AU by 23%, relative to the drained controls; the corresponding values in the WL16 days treatment were 41% in Celera II-AU and 40% in Jade-AU. For Onyx-AU in WL8, root dry matter decreased by 13% during waterlogging (Figure 4B). At the end of the recovery (39 DAS), Jade-AU recovered its root growth, showing similar root dry weight with its control under the shorter duration of waterlogging (WL2) but a 24% reduction in Celera II-AU and 33% in Onyx-AU. Root dry matter had declined by 62% in Celera II-AU, 65% in Jade-AU, and 40% in Onyx-AU in both WL8 and WL16 treatments (Figure 4B).

The reduction of root dry matter resulted from decay and damage to the existing root system. For all genotypes, the maximum depth of the taproot did not significantly differ from the control in the treatments (WL2 and WL4), showing the ability of roots to penetrate WL soil. The taproot even survived 16 days of waterlogging. Jade-AU consistently maintained its maximum taproot length throughout the experiment in all waterlogging treatments (Table 4) but the taproot length of Celera II-AU and Onyx-AU significantly decreased (*P* < 0.001) in the 16 days of waterlogging treatment before recovery.

### Adventitious Root Formation

**Figure 6:**
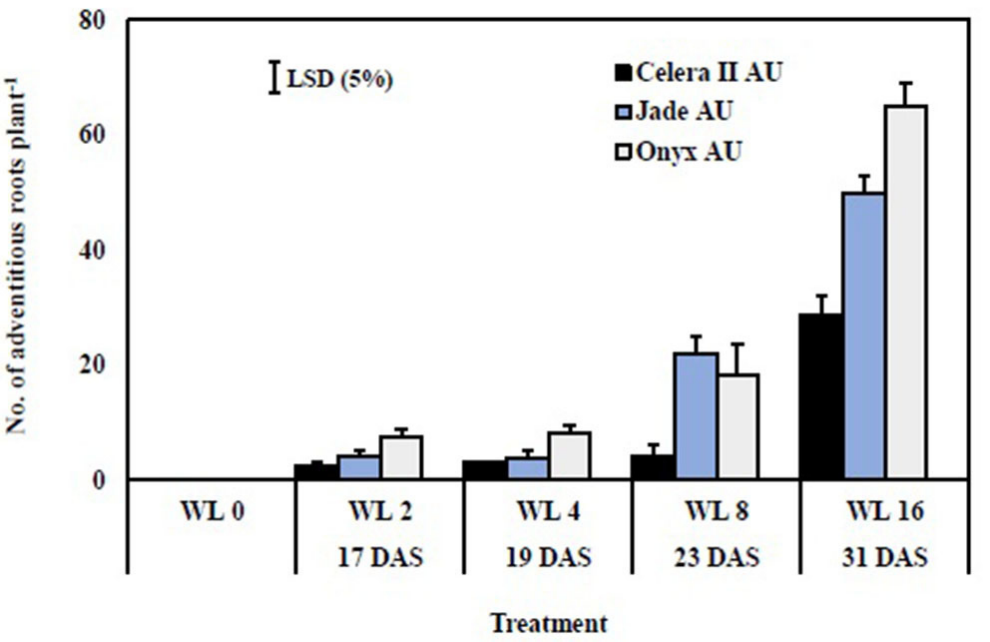
Number of adventitious roots after different waterlogging durations. W0, drained control; WL2, waterlogging for 2 days; WL4, waterlogging for 4 days; WL8, waterlogging for 8 days; WL16, waterlogging for 16 days. Adventitious roots did not form on plants in the drained controls. Harvest occurred at 17 DAS (WL2), 19 DAS (WL4), 23 DAS (WL8), and 31 DAS (WL16). Bars are the mean (*n* = 4) ± SE and LSD at *P* = 0.05 for the genotype.

There were no adventitious roots on plants in the drained controls. Adventitious roots were initiated near the shoot-root junction (hypocotyl region) and ranged in length from 0.2 to 0.5 cm after 2 days of waterlogging. The length and number of adventitious roots increased with the duration of waterlogging and varied with genotype. Adventitious root length reached 3–4 cm on average after WL8 to 7–10 cm after WL16. The effects of waterlogging duration, genotype, and their interaction significantly differed (*P* < 0.001) (Table 3). The genotypes differed in 8 and 16 days of treatments, with Jade-AU and Onyx-AU producing more adventitious roots than Celera II-AU (Figure 6). Furthermore, some surface roots were initiated from the lateral roots while others emanated from adventitious roots near the soil surface after 4 days of waterlogging. However, surface root tips dried up during the recovery stage after draining. By the end of the recovery period, the adventitious roots had resumed their elongation and extended up to 15–20 cm (data not shown).

### Relative Growth Rate

All genotypes had 2–3 times higher root RGR than shoot RGR in the drained control after WL2 treatment (17 DAS). After 19 DAS, Celera II-AU quickly increased its shoot RGR from 0.11 to 0.27 g g^−1^ d^−1^ and decreased its root RGR from 0.31 to 0.03 g g^−1^ d^−1^. Waterlogging stress reduced shoot and root RGRs, relative to the drained controls in all waterlogging treatments, except for Jade-AU in WL2 (Supplementary Figure 3). The shoot RGR of Onyx-AU after 8 days of recovery following WL16 was 0.028 g g^−1^ d^−1^, relative to 0.075 g g^−1^ d^−1^ in the control, resulting in a 40% decline in shoot dry mass. Despite the rapid formation of adventitious roots in the WL8 and WL16 treatments, total root dry matter did not fully recover to the level of the drained control. The roots of Celera II-AU had a negative RGR (−007 g g^−1^ d^−1^) during recovery in the WL16 treatment, compared with 0.036 g g^−1^ d^−1^ in the drained control (Supplementary Figure 4).

### SPAD Chlorophyll Content

**Figure 7:**
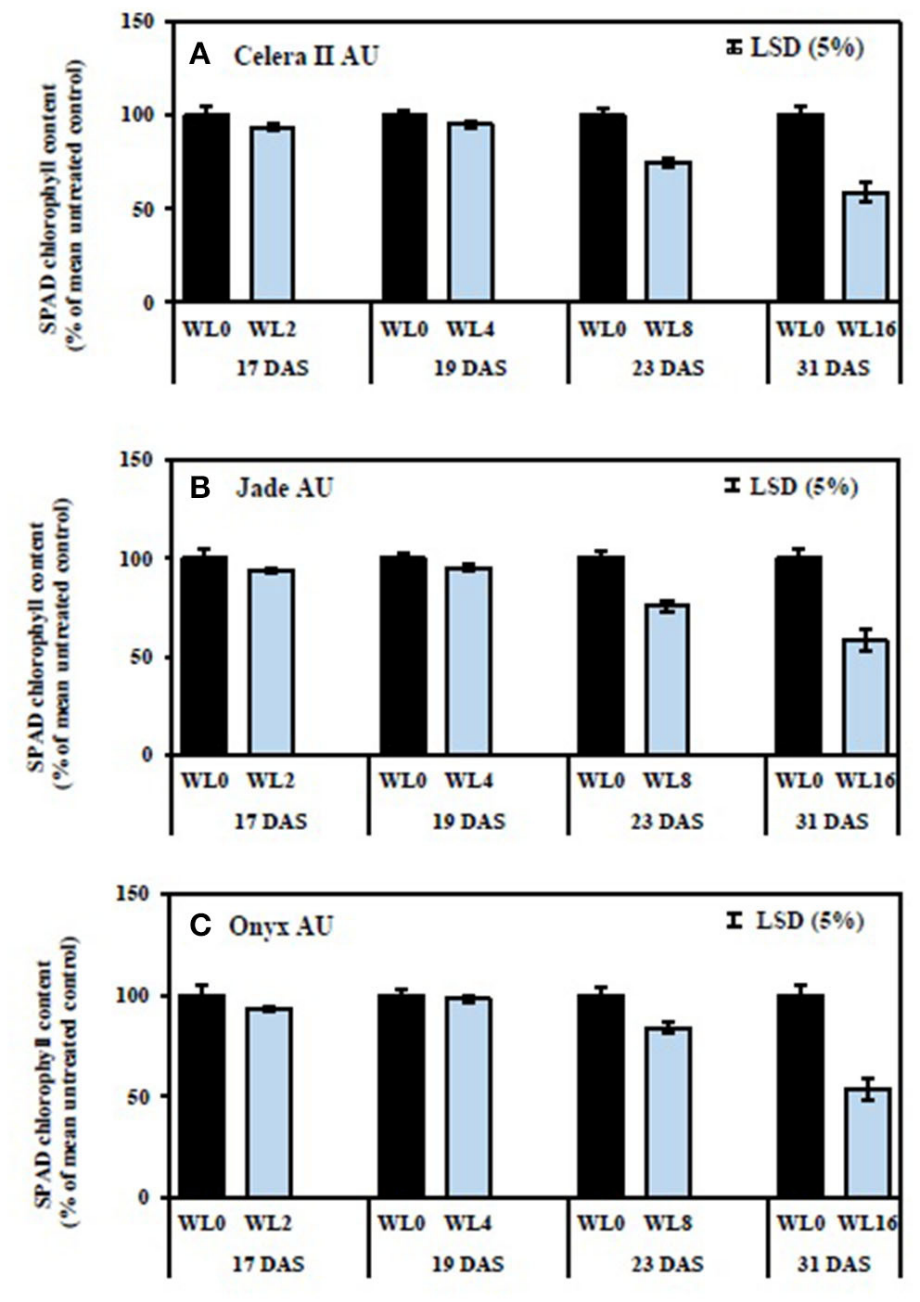
Relative chlorophyll content (SPAD readings of the first trifoliate leaf) under different waterlogging durations for **(A)** Celera II-AU, **(B)** Jade-AU, and **(C)** Onyx-AU. The percentage of SPAD chlorophyll content for WL plants was estimated relative to their drained controls. WL0, drained control; WL2, WL for 2 days, harvested at 17 DAS; WL4, WL for 4 days, harvested at 19 DAS; WL8, WL for 8 days, harvested at 23 DAS; WL16, WL for 16 days, harvested at 31 DAS. The average SPAD chlorophyll content in the drained control is 41 ± 3 in Celera II AU, 50 ± 3 in Jade AU, and 44 ± 3 in Onyx AU. Data are means ± SE of four replicate pots. Vertical bars are LSD at *P* = 0.05 for the genotype.

**Figure 8:**
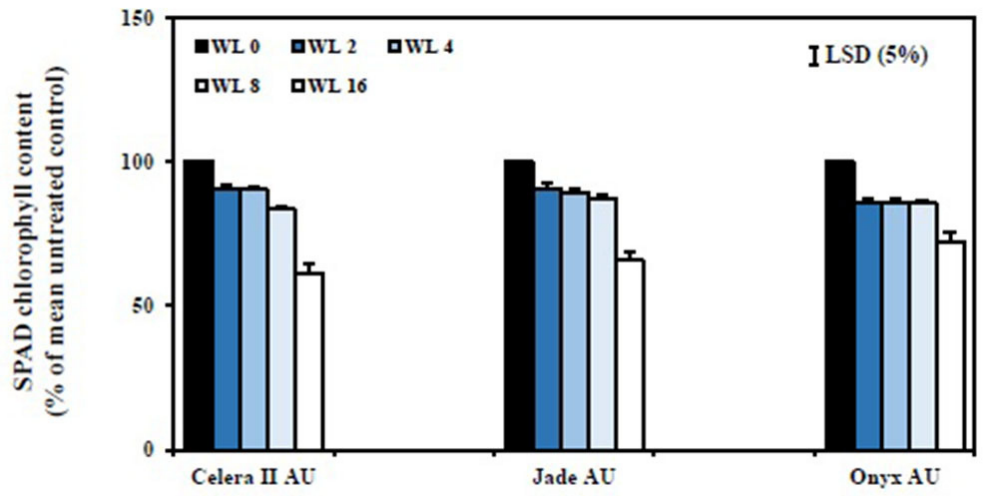
Relative chlorophyll content (SPAD readings of the first trifoliate leaf) of different genotypes at the end of the recovery (39 DAS). The percentage of SPAD chlorophyll content for WL plants was estimated relative to their drained controls. WL0, drained control; WL2, waterlogging for 2 days, recovery for 22 days; WL4, waterlogging for 4 days, recovery for 20 days; WL8, waterlogging for 8 days, recovery for 16 days; WL16, waterlogging for 16 days, recovery for 8 days. Data are means ± SE of four replicate pots. Vertical bars are LSD at *P* = 0.05 for the genotype.

SPAD chlorophyll content values of the first trifoliate leaves were recorded to understand the effect of waterlogging on leaf nitrogen status. Jade-AU had the highest chlorophyll content (SPAD value) in the drained control, followed by Onyx-AU and Celera II-AU. Conversely, waterlogging reduced SPAD chlorophyll content at similar rates for all genotypes. Short-term waterlogging (i.e., up to WL4) had no significant effect on chlorophyll content. The WL8 and WL16 treatments significantly (*P* < 0.001) reduced the overall chlorophyll content, relative to the drained control, for all genotypes (Figure 7). The rate of recovery was affected by waterlogging duration (Figure 8). The WL2 and WL4 treatments returned to the drained control level SPAD chlorophyll, and visually reverted from a pale yellow to green just 2 days after drainage. Genotypic variation for SPAD value was the greatest at recovery in the WL16 treatment (39 DAS), being 72% of the control in Onyx-AU, 66% in Jade-AU, and 61% in Celera II-AU.

### Nodule Formation

In the drained controls, plant nodulation scores increased as nodule number and size increased with crop growth. At the initial harvest (H1) in the drained control, Jade-AU had the highest nodulation score, followed by Onyx-AU, while Celera II-AU had no nodules at this point. Under the different waterlogging durations, all plants produced root nodules near the soil surface (visual observation), which appeared to survive even in the WL16 treatment and continued to grow during the recovery (Tables 4, 5). However, for roots in WL soil, the nodules at depth were white, indicating that they were not functional. No nodules were observed on adventitious roots.

## Discussion

Waterlogging is destructive abiotic stress, the occurrence of which is increasing in some parts of the world due to a high frequency of unseasonal rainfall caused by global climate change. This is the first study to compare mungbean and blackgram genotypes under different waterlogging durations at two critical growth stages, such as germination and seedling stages. The current study found that waterlogging delayed and with longer duration reduced seed germination, seedling emergence, and establishment. Waterlogging at the seedling stage reduced shoot and root dry mass, which was proportional to the waterlogging duration. At the end of the experiment, plants exposed to 16 days of waterlogging and then allowed 8 days recovery had 60% less total dry mass in mungbean and 40% less in blackgram than those of drained control plants. One unanticipated finding was that no seedlings died even after 16 days of waterlogging (almost one-fourth of their life cycle). The plants exhibited adaptation to transient waterlogging through extensive adventitious root formation. The effect of soil waterlogging on seed germination and seedling growth varied between the genotypes, as discussed below.

At germination, the drained controls had rapid seedling emergence starting at 3 DAS and completed full emergence (100%) at 6–7 DAS. During soil waterlogging, all three genotypes exhibited no seedling emergence. These seeds in WL soil presumably could not germinate due to a lack of sufficient oxygen. When the pots with shorter durations of waterlogging were drained, some seedlings then emerged, but with longer durations of waterlogging the seeds had lost viability, as evidenced by the failure to germinate and establish seedlings upon drainage of the previously WL pots. Some seeds grew hypocotyls out of their testa, but no growth beyond this was observed, and the seeds failed to emerge after 8 days of waterlogging. Hence, the longer the waterlogging duration, the greater the reduction in germination and seedling establishment. Previous research indicates that in other legumes [i.e., common bean (*Phaseolus vulgaris* L.) and soybean] there is a failure to emerge under soil-WL conditions because low oxygen levels restrict the respiration process required for germination (Morinaga, 1926; Cardwell, 1984; Hou and Thseng, 1992; Tian et al., 2005; Rajashekar and Baek, 2014). The prolonged period of anaerobiosis by soil waterlogging also results in the death of germinating seeds in waterlogging sensitive peas (Zaman et al., 2018). Similarly, other dryland crops such as wheat and barley could not germinate under WL conditions due to lack of amylolytic enzymes, and rapid uptake of excessive water by seeds led to membrane damage and solute leakage (Powell and Matthews, 1978).

The tolerance of crops to waterlogging may vary depending on plant species. Recently, two contrasting tolerance mechanisms were studied in response to transient waterlogging in field pea, such as “escape” (germination under waterlogging) and “quiescence” (germination/emergence only after the removal of the stress) (Zaman et al., 2018). Another important finding in the seeds of soybean is that the aleurone layer helps to block the abrupt water entry into the embryo, as it covers the surface of the embryo, absorbing water slowly and maintaining membrane integrity under transient waterlogging (Tian et al., 2005). Furthermore, seed testa color appears to be related to waterlogging tolerance, with dark testa genotypes more tolerant than light testa genotypes in wheat (Ueno and Takahashi, 1997), soybean (Hou and Thseng, 1991), and field peas (Zaman et al., 2019). In the present study, that blackgram (dark testa and hypocotyl pigmentation) has higher seedling emergence upon drainage following a period of waterlogging than mungbean (green testa) (Figure 2). Of the two mungbean genotypes, Celera II (small-seeded and hypocotyl pigmentation) had a better seedling establishment than Jade-AU (large-seeded and no hypocotyl pigmentation) under waterlogging. To develop a full picture of waterlogging tolerance at the germination stage, additional studies on diverse germplasm are needed to reveal possible tolerance mechanisms in both species.

In the seedling stage, the formation of adventitious roots probably played an important role in waterlogging tolerance. Sauter (2013) reported that adventitious roots, with aerenchyma which facilitates oxygen movement within these roots, enable water and nutrient uptake through these roots, and thus plant survival and even growth. Adventitious roots also influenced the soil redox potential in this study. At the start of waterlogging treatment, the soil redox potential under waterlogging at the seedling stage declined as in the germination trial, stabilizing after 5 days. However, by Day 7, the soil redox potential started to trend upwards presumably because the seedlings started to produce adventitious roots under waterlogging, and there would likely have been some radial oxygen loss from those roots to the soil (Armstrong, 1979). No such roots were observed in the drained controls. Hence, adventitious roots formed at the seedling stage under waterlogging are regarded as an adaptation in both mungbean and blackgram under waterlogging.

Adventitious root formation is a quantitative, heritable trait controlled by multiple factors, including species, genotype, growth stage, water temperature, and waterlogging duration and depth (Lorbiecke and Sauter, 1999; Sorin et al., 2006; Dawood et al., 2014; Argus et al., 2015; Zhang et al., 2015). Various studies have assessed the efficacy of waterlogging stimulated adventitious roots containing aerenchyma as an adaptive response (Shimamura et al., 2003; Visser and Voesenek, 2004; Thomas et al., 2005; Sauter, 2013; Steffens and Rasmussen, 2016). In addition, root growth-regulating hormones might be important in the formation of aerenchymatous adventitious roots [e.g., rice (Lin and Sauter, 2020), Rumex (Visser et al., 1996), wheat (Nguyen et al., 2018), and Arabidopsis (Verstraeten et al., 2014)]. Nonetheless, formation of aerenchyma in response to waterlogging varies depending on plant species (Smirnoff and Crawford, 1983; Justin and Armstrong, 1987; Visser et al., 2000; McDonald et al., 2002; Grimoldi et al., 2005). Yamauchi et al. (2013) reported the role of primary aerenchyma in the adventitious roots of cereals (rice, maize, barley, and wheat) and secondary aerenchyma in the stem, hypocotyl, taproot, adventitious roots, and root nodules of legumes such as soybean. Further studies are required to understand the physiological and genetic bases of the formation of adventitious roots, the role of aerenchyma inside adventitious roots, and root porosity in mungbean and blackgram under soil waterlogging stress.

Waterlogging reduced root and shoot dry matter by damaging the existing root systems of mungbean and blackgram (current study) as shown also for some other crop species (wheat, Malik et al., 2002; chickpea and fababean, Munir et al., 2019). Although seedlings responded to transient waterlogging by producing adventitious roots and large nodules on the primary root near the soil surface, however, that did not compensate for the role of the roots that were formed at the initial stage. Damage to the root systems under waterlogging had consequences for the shoots, such as a reduced number of leaves and a smaller leaf area. Limitation of nutrient uptake, as likely demonstrated for N by reduced chlorophyll content (SPAD value) following a longer duration of waterlogging (WL8 and WL16), could be one cause of the reduction in shoot growth. Adverse effects of waterlogging on nutrient acquisition have been reported previously [e.g., soybean (Bacanamwo and Purcell, 1999); wheat (Malik et al., 2002); soybean (Board, 2008); pea, lentil, and grass pea (Malik et al., 2015); cotton (Najeeb et al., 2015)]. The reasons behind these reductions could be due to the death and decay of most lateral roots and the incomplete capacity of the adventitious roots to fully compensate for the loss of the other roots.

Another important consideration is the role of nodules housing rhizobia in the roots of legumes in WL soils. In legumes, symbiotic nitrogen fixation through rhizobia nodules is the main source of nitrogen acquisition. In the present study, mungbean and blackgram in the drained controls produced more nodules than the WL treatments (Tables 4, 5). Nodules below 5 cm of WL soil might have become ineffective after 4 days of waterlogging as their color changed from pink to white (observed by cutting open nodules). Nodules at the plant crown continued to grow under different waterlogging durations and presumably were able to function into the recovery stage, as these were always pink. Similar nodulation was observed in a tropical forage legume, American jointvetch (*Aeschynomene americana* L.), which maintained nitrogenase activity and net assimilation rate for growth under WL conditions (Tobisa et al., 2014). Previous research indicated that legumes could change the pathway of oxygen diffusion to nodules (Roberts et al., 2010). In soybean, studies have shown that the aerenchyma connects to the outer cortex of nodules, presumably enabling their functioning for roots in WL soils (Shimamura et al., 2003; Thomas et al., 2005).

The genotypes differed in responses to the waterlogging treatments at both growth stages (significant statistical interaction in ANOVA; Table 3). Genotypic variation in waterlogging tolerance was greatest at the germination stage after 4 days of waterlogging and at the seedling stage after 8 days of waterlogging. Blackgram, Onyx-AU, had a higher germination rate and seedling establishment than the mungbean genotypes for all waterlogging durations. Genotypic variation was evident for adventitious root formation in plants WL at the seedling stage. Onyx-AU and Jade-AU rapidly produced many adventitious after 8 days of waterlogging, with less extensive growth in Celera II-AU (Supplementary Figure 4). Onyx-AU, a blackgram genotype, was more tolerant to transient waterlogging than Jade-AU and Celera II-AU in both growth stages. Between the two mungbeans, Celera II-AU had a higher germination percent than Jade-AU. In contrast, Jade-AU grew more and recovered quicker after waterlogging at the seedling stage than Celera II-AU. Further research should be undertaken to identify the linkage between waterlogging tolerance and morphological traits, e.g., seed size, hypocotyl pigmentation, and testa color in the two species.

The results of this research have significant implications for the understanding of waterlogging tolerance in mungbean and blackgram. As genetic tolerance would enhance yield and its stability, there is a need for systematic screening of a wide range of germplasm to identify and exploit the genetic variation in both species. The methodology developed in this study can be used for the design of extensive screening of mungbean mini-core germplasms to identify waterlogging tolerance and to understand its genetic basis. This is a preliminary study of the tolerance of mungbean and blackgram at the germination and seedling stages. Further research can now be conducted to identify tolerance in both species to transient waterlogging using a larger number of genotypes.

## Conclusion

This research compared mungbean and blackgram cultivars under different waterlogging durations at two critical growth stages, such as (i) germination and (ii) early vegetative growth. All waterlogging treatments significantly reduced germination and retarded seedling growth; adverse effects were greater with longer waterlogging duration. Prolonged waterlogging duration adversely affected the germination rate, plant establishment, shoot, and root growth and development. Onyx-AU (blackgram) can cope with the low-oxygen environment than Jade-AU and Celera II-AU (mungbean) in both growth stages. To follow an adaptive strategy at the seedling stage, waterlogging for 16 days radically reduced growth but did not result in plant death due to plant adaptations to stress, with the help of producing adventitious roots and rhizobia nodules near the crown.

## Data Availability Statement

The original contributions presented in the study are included in the article/Supplementary Materials, further inquiries can be directed to the corresponding author/s.

## Author Contributions

KK performed the experiments. KK, AM, TC, KS, and WE designed the experiments, contributed to the analysis, and wrote and edited the manuscript. All authors contributed to the article and approved the submitted version.

## Conflict of Interest

The authors declare that the research was conducted in the absence of any commercial or financial relationships that could be construed as a potential conflict of interest.

## Publisher's Note

All claims expressed in this article are solely those of the authors and do not necessarily represent those of their affiliated organizations, or those of the publisher, the editors and the reviewers. Any product that may be evaluated in this article, or claim that may be made by its manufacturer, is not guaranteed or endorsed by the publisher.

## Supplementary Material

Supplementary Figure 1 Effect of waterlogging on soil redox potential at the germination stage in the waterlogged (WL) treatments, relative to the drained control. Error bars represent standard errors of the mean.

Supplementary Figure 2 Comparison of total leaf area between drained control and WL plants for three genotypes after **(A)** different waterlogging duration and **(B)** recovery. WL0, drained control; WL2, waterlogging for 2 days, recovery for 22 days; WL4, waterlogging for 4 days, recovery for 20 days; WL8, waterlogging for 8 days, recovery for 16 days; WL16, waterlogging for 16 days, recovery for 8 days. Bars are means ± SE of four replicates. Least significant differences (LSD) at *P* = 0.05 for the genotype.

Supplementary Figure 3 Shoot relative growth rate (RGR) under different waterlogging durations and subsequent recovery for **(A)** Celera II-AU, **(B)** Jade-AU, **(C)** Onyx-AU. WL0, drained control; WL2, waterlogging for 2 days, recovery for 22 days; WL4, waterlogging for 4 days, recovery for 20 days; WL8, waterlogging for 8 days, recovery for 16 days; WL16, waterlogging for 16 days, recovery for 8 days. RGR is estimated from the mean of four replicate pots.

Supplementary Figure 4 Root RGR under different waterlogging durations and subsequent recovery for **(A)** Celera II-AU, **(B)** Jade-AU, **(C)** Onyx-AU. WL0, drained control; WL2, waterlogging for 2 days, recovery for 22 days; WL4, waterlogging for 4 days, recovery for 20 days; WL8, waterlogging for 8 days, recovery for 16 days; WL16, waterlogging for 16 days, recovery for 8 days. RGR is estimated from the mean of four replicate pots.
