# Supplementary figures and images for: Response of Mungbean (cvs. Celera II-AU and Jade-AU) and Blackgram (cv. Onyx-AU) to Transient Waterlogging
Source: Front Plant Sci. 2021 Aug 19;12:709102. doi: 10.3389/fpls.2021.709102 (PMC8417111; doi:10.3389/fpls.2021.709102)

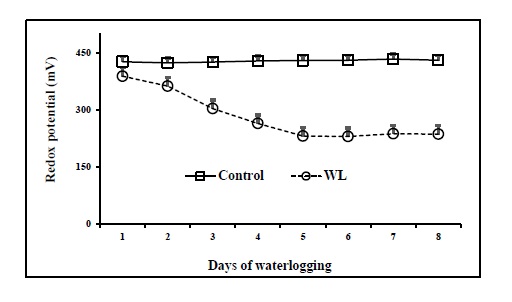

Supplement: Supplementary Figure 1 — Effect of waterlogging on soil redox potential at the germination stage in the waterlogged (WL) treatments, relative to the drained control. Error bars represent standard errors of the mean. [file Image_1.JPEG]

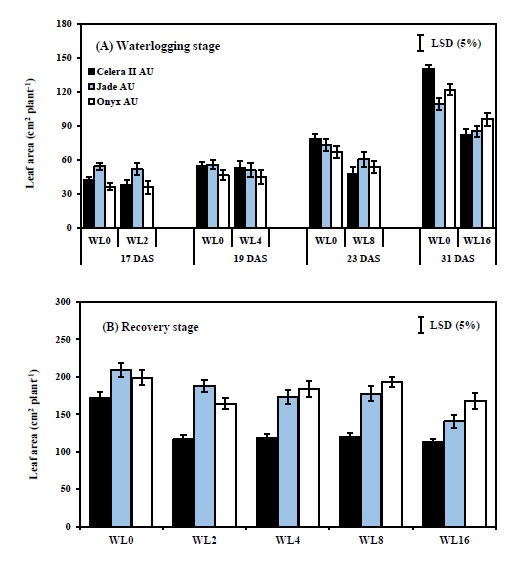

Supplement: Supplementary Figure 2 — Comparison of total leaf area between drained control and WL plants for three genotypes after (A) different waterlogging duration and (B) recovery. WL0, drained control; WL2, waterlogging for 2 days, recovery for 22 days; WL4, waterlogging for 4 days, recovery for 20 days; WL8, waterlogging for 8 days, recovery for 16 days; WL16, waterlogging for 16 days, recovery for 8 days. Bars are means ± SE of four replicates. Least significant differences (LSD) at P = 0.05 for the genotype. [file Image_2.JPEG]

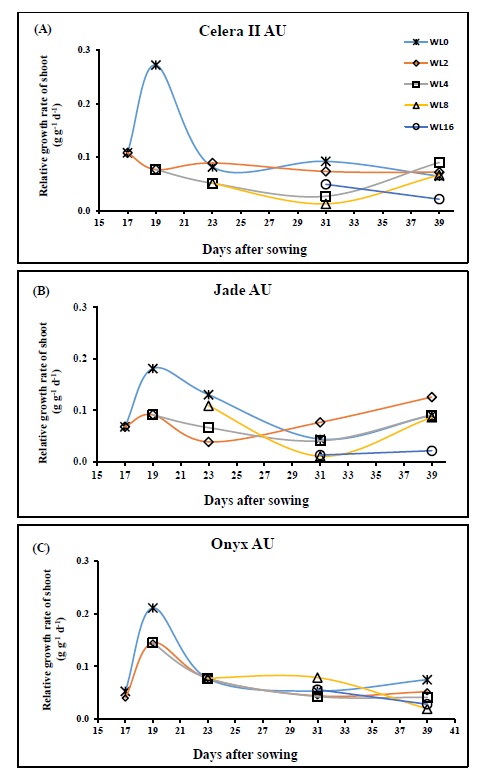

Supplement: Supplementary Figure 3 — Shoot relative growth rate (RGR) under different waterlogging durations and subsequent recovery for (A) Celera II-AU, (B) Jade-AU, (C) Onyx-AU. WL0, drained control; WL2, waterlogging for 2 days, recovery for 22 days; WL4, waterlogging for 4 days, recovery for 20 days; WL8, waterlogging for 8 days, recovery for 16 days; WL16, waterlogging for 16 days, recovery for 8 days. RGR is estimated from the mean of four replicate pots. [file Image_3.JPEG]

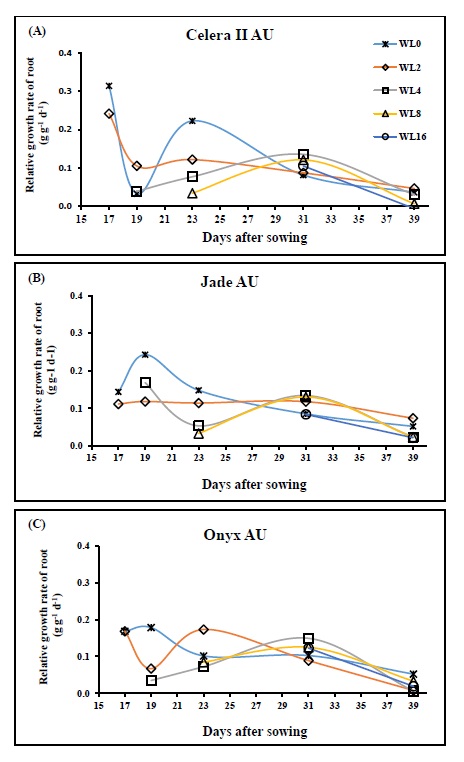

Supplement: Supplementary Figure 4 — Root RGR under different waterlogging durations and subsequent recovery for (A) Celera II-AU, (B) Jade-AU, (C) Onyx-AU. WL0, drained control; WL2, waterlogging for 2 days, recovery for 22 days; WL4, waterlogging for 4 days, recovery for 20 days; WL8, waterlogging for 8 days, recovery for 16 days; WL16, waterlogging for 16 days, recovery for 8 days. RGR is estimated from the mean of four replicate pots. [file Image_4.JPEG]
